# Commendation

**Published:** 1914-05

**Authors:** 


### Commendation.

The recommendations of the commission are timely. They are notable for the occlusion of the old worn-out, futile efforts at segregation and regulation. It declines making society a partner in the business of legalized immorality. It clearly points out the most important factor in the promotion of vice and recommends practical measures whereby it may be largely suppressed. It sees the importance of heredity as a cause and approves of the practice of sterilization as the (only) means to weed out the inborn moral character, which at bottom is the real cause of vice.

Most commendable of all is the suggestion which provides for the hospital care of venereal cases, and their theatment as contagious diseases. It is evident to all that secrecy more than ignorance is responsible for the greater prevalence of vice. The time has come when these affections must be regarded as a menace to public health, and to stop their ravages they must be cured, as promptly and efficiently as possible.

That this condition may be made practical there must be a change in the public attitude towards venereal infection. While not condoning immoral practices, yet, recognizing such as a condition actually existing, and until this is eliminated, the evil effect on the health of the community must be safeguarded by a more sane humanitarian aspect towards the whole question.

